# Impact of Magnetic Fields on Arc Pressure, Temperature, Plasma Velocity, and Voltage in TIG Welding

**DOI:** 10.3390/mi16090967

**Published:** 2025-08-22

**Authors:** Gang Chen, Gaosong Li, Lei Wu, Zhenya Wang

**Affiliations:** 1 School of Mechanical and Electric Engineering, Sanming University, Sanming 365004, China; chengang@fjsmu.edu.cn (G.C.); wulei@fjsmu.edu.cn (L.W.); 2 School of Intelligent Manufacturing Institute, HuangHuai University, Zhumadian 463000, China; 3 Department of Mechanical Engineering, Tsinghua University, Beijing 100084, China

**Keywords:** longitudinal magnetic field, TIG welding arc, temperature, pressure field, plasma velocity

## Abstract

A longitudinal magnetic field provides a new method for regulating the plasma velocity, pressure field, and temperature field of the TIG welding arc. However, the mechanism of action of the longitudinal magnetic field remains poorly understood. In order to address this problem, this paper develops a numerical model based on continuum mechanics. The mechanism of how magnetic field strength affects temperature, pressure field, plasma velocity, and potential was investigated. The geometric shape, temperature, pressure, and plasma velocity of the TIG welding arc under different magnetic fields were predicted. The results indicate that as magnetic field strength increases, the arc shape is compressed under the influence of magnetic forces, with the degree of compression increasing with magnetic field strength; plasma velocity gradually increases from 74 m/s at 0 mT to 296 m/s at 150 mT, but the velocity along the arc’s central axis first decreases and then increases with increasing magnetic field strength. As the magnetic field strength increases, a negative pressure first appears near the cathode, then expands toward the cathode, and finally toward the anode. During the expansion of the negative pressure, the maximum absolute value of the arc pressure increases by 12.72 times.

## 1. Introduction

TIG welding is a kind of tungsten inert gas with the advantages of precise heat input control, high weld quality, and a wide range of material applications [1,2,3]. Compared with metal inert gas (MIG) welding, shielded metal arc welding (SMAW) and submerged arc welding (SAW), it has the advantages of low impurities, less heat input, and excellent mechanical properties, and is widely used in aerospace, petrochemical, precision instruments, and automotive transportation, etc. [4,5,6]. TIG welding technology is not only capable of realising the connection of homogeneous metals but also heterogeneous metals under the premise of selecting suitable fillers [7]. However, during TIG welding, the morphology, temperature, pressure, and plasma velocities of the welding arc have an important influence on mechanical properties of welding seams [8,9,10]. Most current studies on TIG welding rely primarily on experimental measurements [11]. Specifically, these investigations examine the effects of arc morphology, temperature, and pressure on weld chemical properties by analysing the microstructure, composition, and tensile properties of welded workpieces. However, this methodology entails inherent limitations and uncertainties [12].

Numerical calculation breaks the shortcomings of traditional experiments, as it is difficult to observe the arc temperature, plasma speed, and pressure, etc., and has a positive role in promoting the development of TIG welding technology. Arora et al. developed and assembled a 3D model of TIG welding using SolidWorks 2025 and studied temperature changes [13]. Song et al. established a two-dimensional numerical model of K-TIG plasma and studied the effect of welding parameters on arc characteristics. The results showed that the temperature gradually decreased as the arc moved away from the arc centre [14]. Li et al. established a magnetohydrodynamic model of a high-current vacuum arc and investigated the effect of the arc transverse magnetic field on the distribution of the plasma, and the result was that the surface magnetic field could change the arc’s symmetry [15]. Qin et al. established a three-dimensional transient moving heat source numerical model for TIG welding, simulated the temperature field, and provided effective guidance for optimising process parameters [16]. Yadav established a numerical model for thermal stress and predicted the peak temperature during welding [17]. Aberbache et al. developed a numerical model for TIG welding that can predict the temperature field, predicted changes in temperature distribution, and conducted experimental verification [18]. Ebrahimpour et al. developed a mathematical model to study the effect of angle on the heat source in TIG-MIG, investigated the influence of arc configuration and welding gun angle on the thermal distribution of the arc, and validated the correctness of the model [19]. Lei et al. developed a two-dimensional model of the arc plasma, studied the changes in temperature field, pressure field, and plasma velocity of hollow and solid tungsten electrodes, and validated the model experimentally [20].

The above-mentioned studies have made important contributions to the advancement of tig welding technology. However, most of the studies did not consider the impact of longitudinal magnetic field on arc morphology, temperature, pressure, and plasma velocity, which limits the adjustable range of the TIG welding process. For example, Wei et al. developed a numerical model of magnetohydrodynamics to investigate the effect of different gaps on the arcing problem and verified it experimentally [21]. Meanwhile, most of the existing studies assumed that the thermophysical property parameters of the welding materials are constants. For instance, Tang et al. established a temperature field model with constant thermophysical properties of materials and used this model to study the effect of materials on the arc temperature field. The results showed that there were significant differences in arc temperatures between different materials [22]. In addition, few existing studies have investigated the central axis and the variation in plasma velocity off the central axis both from the perspective of electric field gradient and magnetic field force. For example, Zong developed a multi-physics coupled numerical model of the HTM arc, but the model did not describe the mechanism of magnetic field action within plasma [23]. Therefore, the establishment of a TIG welding model containing longitudinal magnetic field is of great theoretical and applied value for the study of TIG welding morphology, temperature, pressure and plasma velocity regulation.

This paper establishes a multi-physics TIG welding numerical model incorporating a longitudinal magnetic field to investigate the mechanisms by which the magnetic field influences the plasma velocity, pressure field, and temperature field of the TIG welding arc. The model accounts for the effects of temperature-dependent, thermal-physical shape and arc physical properties. Temperature fields, pressure fields, plasma velocities, and arc morphologies under different magnetic fields are predicted, and experimental validation is conducted based on the arc temperature field morphology. The study investigates the influence of magnetic fields on arc temperature, pressure, and plasma velocity. It elucidates the intrinsic reasons why the high-temperature region of the arc is compressed as magnetic field strength increases and moves away from the arc’s central axis. Additionally, it explains why, as magnetic field strength increases, negative pressure first appears near the cathode, initially expanding toward the cathode and then toward the anode.

## 2. Numerical Model

### 2.1. Models and Assumptions

**Figure 1:**
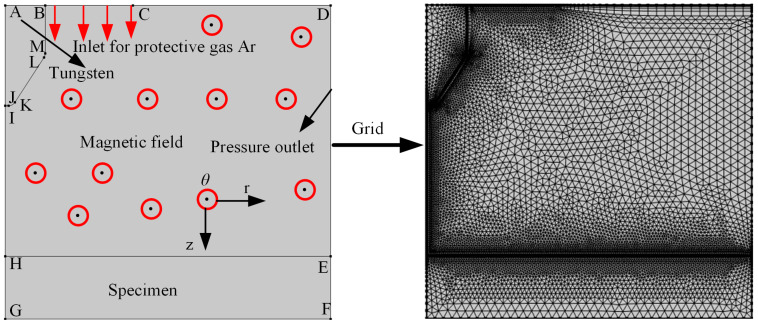
Numerical model and mesh.

The numerical model of the tungsten arc welding arc is illustrated in Figure 1. Tungsten electrodes have a diameter and length of 1.6 mm and 4 mm, respectively, and the tip of the tungsten electrode is rounded, with a distance of 6 mm from the welding plane. The magnetic field in this model is a longitudinal magnetic field, and the current is 200 A. Only the radial component of the current generates a magnetic force. Therefore, in COMSOL 6.0, the electromagnetic force is added to the model in the form of a volume force in laminar flow, with a magnitude of −B1∗mef.Jr. The magnetic field strength is scanned from 0 mT, with a scan interval of 1 mT and a maximum scan value of 1200 mT.

The maximum unit at the boundary is 0.0838 mm, with a boundary layer count of 6. The mesh type employed is triangular, and the protective gas for the tungsten electrode is high-purity argon. The model is developed in the COMSOL Multi-physics software platform and executed on a PC with Intel Core i7-9700, 3.00 GHz, 56 GB RAM and Windows 10 system. Numerical model of “Impact of magnetic fields on arc pressure, temperature, plasma velocity, and voltage” in TIG welding required 1 h to complete the calculations. The capital letters in Figure 1 indicate boundary points. To simplify models, the following assumptions have been made as follows:

1. The arc is assumed to be in steady state and axisymmetric in two dimensions;
2. The effect of the anode surface state on the arc is not considered;
3. The plasma isoforms of the arc are laminar;
4. The external environment has standard atmospheric pressure, and the physical properties of argon gas depend on temperature;
5. A uniform current density is assumed at the tip of the tungsten electrode.

### 2.2. Grid Independence Verification

**Figure 2:**
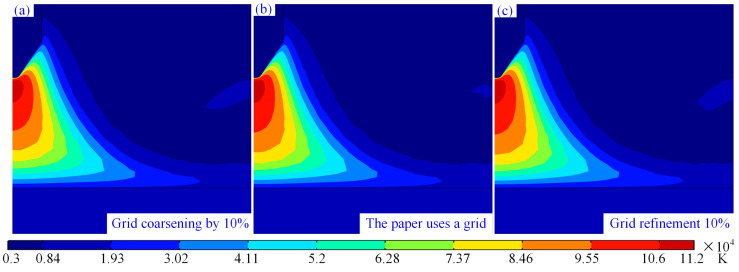
Research on grid independence: (**a**) maximum grid of 0.09218 mm, (**b**) maximum grid of 0.0838mm, and (**c**) maximum grid of 0.07542 mm.

Figure 2 shows the temperature distribution cloud maps of the arc under three different grid settings, where the minimum cell size in Figure 2b is 0.00025 mm and the maximum cell size is 0.0838 mm. The maximum and minimum cell sizes in Figure 2a are 10% larger than those in Figure 2b, while those in Figure 2c are 10% smaller than those in Figure 2b. From Figure 2a–c, it can be observed that although there are slight differences in the temperature field of the arc under the three different grid configurations, the differences are minimal. This indicates the independence of the selected minimum cell size of 0.00025 mm and maximum cell size of 0.0838 mm in this study.

### 2.3. Control Equation

The set of control equations for solving the two-dimensional axisymmetric arc model includes mass, momentum and energy conservation equations. The mass conservation equation is shown in (1) [24].

(1) $$\frac{\partial \rho }{\partial t}+\frac{1}{r}\frac{\partial \left(r\rho v\right)}{\partial r}+\frac{\partial \left(\rho u\right)}{\partial z}=0$$

Then, the conservations of the radial and axial momentum are presented in (2) and (3), respectively [25].

(2) $$\frac{\partial \left(\rho v\right)}{\partial t}+\frac{1}{r}\frac{\partial \left(r\rho v^{2}\right)}{\partial r}+\frac{\partial \left(\rho uv\right)}{\partial z}=F_{r}-\frac{\partial P}{\partial r}+\frac{1}{r}\frac{\partial }{\partial r}\left(2\mu r\frac{\partial u}{\partial r}\right)-2\mu \frac{v}{r^{2}}+\frac{\partial }{\partial z}\left(\mu \frac{\partial v}{\partial z}+\mu \frac{\partial u}{\partial r}\right)$$

(3) $$\frac{\partial \left(\rho u\right)}{\partial t}+\frac{1}{r}\frac{\partial \left(r\rho uv\right)}{\partial r}+\frac{\partial \left(\rho u^{2}\right)}{\partial z}=F_{z}-\frac{\partial P}{\partial z}+\frac{1}{r}\frac{\partial }{\partial r}\left(\mu r\frac{\partial v}{\partial z}+\mu r\frac{\partial u}{\partial r}\right)+\frac{\partial }{\partial z}\left(2\mu \frac{\partial u}{\partial z}\right)+F_{lorent}$$

After, the conservation of energy can be written as (4) and (5) [26].

(4) $$\frac{\partial \left(\rho c_{p}T\right)}{\partial t}+\frac{1}{r}\frac{\partial \left(r\rho vc_{p}T\right)}{\partial r}+\frac{\partial \left(\rho uc_{p}T\right)}{\partial z}=\frac{1}{r}\frac{\partial }{\partial r}\left(kr\frac{\partial T}{\partial r}\right)+\frac{\partial }{\partial z}\left(k\frac{\partial T}{\partial z}\right)+Q$$

(5) $$F_{lorent}=qVB_{1}=ma$$

In order to solve the physical quantities of the electromagnetic field, it is necessary to introduce the current continuity equation, Ohm’s law, and Ampere’s circulation theorem, as shown in (6)–(8).

(6) $$\frac{\partial }{\partial z}\left(\sigma \frac{\partial \phi }{\partial z}\right)+\frac{1}{r}\frac{\partial }{\partial r}\left(\sigma r\frac{\partial \phi }{\partial r}\right)=0$$

(7) $$J_{r}=-\sigma \frac{\partial \phi }{\partial r},J_{z}=-\sigma \frac{\partial \phi }{\partial z}$$

(8) $$B=\frac{\mu_{0}}{r}\int_{0}^{r}J_{z}rdr$$

### 2.4. Boundary Conditions

The heat loss by arc radiation is denoted by $S_{R}=C_{0}T_{R}^{4}$ [24], where *C*_0_ is the radiation coefficient. The source term *Q* of the energy equation is shown in (9).

(9) $$Q=\frac{J_{z}^{2}+J_{r}^{2}}{\sigma }+\frac{{5k}_{B}}{2e}\left(J_{z}\frac{\partial T}{\partial z}+J_{r}\frac{\partial T}{\partial r}\right)-S_{R}$$

## 3. Results

### 3.1. Longitudinal Magnetic Field Effects on Temperature Field

**Figure 3:**
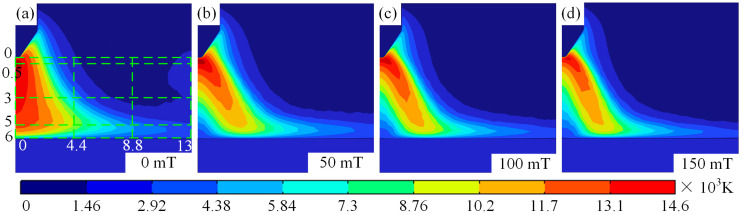
Arc morphology and temperature under different magnetic fields: (**a**) magnetic field of 0 mT, (**b**) magnetic field of 50 mT, (**c**) magnetic field of 100 mT, and (**d**) magnetic field of 150 mT.

During TIG welding, external magnetic fields alter the temperature and morphological characteristics of the arc significantly. To investigate the mechanism of magnetic field influences on arc temperature, Figure 3 displays cloud diagrams of arc temperature distributions under different magnetic fields. As illustrated in Figure 3a–d, the distribution of arc temperature transitions from a bell-shaped configuration to a hollow form, with the increase in magnetic intensity. During TIG welding, applying an external magnetic field can significantly alter the temperature distribution and morphological characteristics of the arc. To investigate the mechanism by which the magnetic field influences the arc temperature field, Figure 3 shows the temperature field distribution contour plots of the arc under different magnetic fields. As shown in Figure 3a–d, as the magnetic field strength increases, the distribution of the arc temperature field gradually changes from a bell shape to a hollow shape. This phenomenon is primarily influenced by two factors. First, magnetic field force: As the magnetic field strength increases, the magnetic field force acting on the plasma deviates it from the arc’s central axis, causing the plasma to move radially along the arc. The farther the plasma is from the cathode, the greater its deviation from the central axis. Second, protective gas cooling and purging: Since the protective gas inlet is in a certain distance from the arc’s central axis, as the plasma moves away from the central axis under the influence of magnetic forces, the compressive force exerted by the protective gas increases as the distance between the central axis and the protective gas inlet axis decreases. Simultaneously, as the plasma moves away from the central axis, the pressure it experiences and the amount of heat lost both increase. Therefore, the arc shape is compressed under the combined effects of magnetic field forces and protective gas, with the degree of compression increasing as the magnetic field strength increases, as shown in Figure 3.

It is worth noting that in TIG welding, regardless of the presence of a magnetic field, the temperature near the cathode is high with a large temperature gradient, while the temperature near the anode is low with a smaller temperature gradient. When the TIG welding magnetic field is 0 mT, arc temp decreases gradually from the centre to the surroundings, and the maximum temperature is 14,600 K, as illustrated in Figure 3a. With increasing magnetic field, the high-temperature area at the arc centre moves away from the central axis under the influence of magnetic force, and maximum arc temperature decreases to 13,500 K with increasing magnetic, as shown in Figure 3a–c. The occurrence of this phenomenon under the influence of a magnetic field on argon arc welding may be attributed to electromagnetic forces. During magnetic field-assisted TIG welding, electromagnetic forces deflect the plasma toward the arc periphery, where the temperature depends on plasma behaviour. As a result, when TIG welding is subjected to a longitudinal magnetic force, high-temperature zones move from the arc centre to edge.

**Figure 4:**
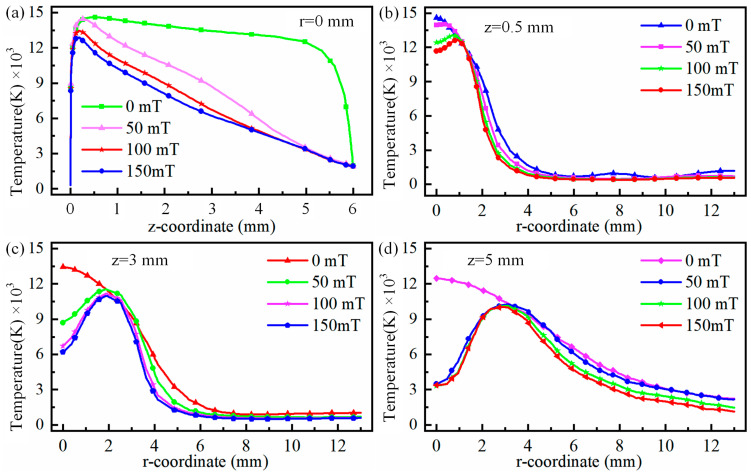
Temperature below tungsten electrode at 0.5 mm, 3 mm, 5 mm and on arc centre axis for different magnetic fields: (**a**) r = 0 mm, (**b**) z = 0.5 mm, (**c**) z = 3 mm, and (**d**) z = 5 mm.

It is worth mentioning that the maximum arc temperature changes from 0.5 mm to 0.13 mm when auxiliary magnetic field rises from 0 mT to 150 mT. Meanwhile, the high-temperature region is shifted to arc edge with the rise in the auxiliary magnetic field, and the maximum shift occurs when auxiliary magnetic field is 150 mT, with a maximum shift distance of 0.45 mm, as illustrated in Figure 3d. Figure 4 displays temperature distributions at 0.5 mm, 3 mm, and 5 mm below the tungsten pole and along the arc centre axis with and without the auxiliary magnetic field. Figure 4a illustrates that the temperature rises rapidly from the cathode to the anode centre axis, and then decreases slowly, irrespective of whether a longitudinal magnetic field has been applied to TIG welds or not. At a magnetic field strength of 0 mT, a maximum temperature of 14,600 K was observed at 0.5 mm below the tungsten electrode.

Then the temperature decreases gradually to 12,000 K between 0.5 mm and 5 mm, then the temperature decreases rapidly to 2000 K after exceeding 5 mm. In TIG welding without an auxiliary field, arc temperatures at the 0.5 mm, 3 mm, and 5 mm positions below tungsten electrode decrease from centre outwards. In addition, the temperature decreases with the distance from the tungsten electrode to the anode centre. Meanwhile, the rate of descent gradually slows down as the distance from the tungsten electrode increases, as shown in Figure 4b,c. The temperature decreases gradually from central values of 14,600 K, 13,344 K, and 12,465 K, slowly declining to 9070 K, 11,298 K, and 11,432 K at a distance of 2 mm and then further decreasing to 1188 K, 1042 K, and 2248 K at 13 mm. In the arc temperature cloud map, the centre of the depression is caused by electrons gathering at the tip. The greater density of electrons, the more the generation of Joule heat, and the higher the arc temperature. There are two reasons for the depression centre: first, electrons gather at the tip, and the greater density of electrons, the more Joule heat, and the higher the arc temperature; second, the cooling and purging effect of the protective gas.

**Figure 5:**
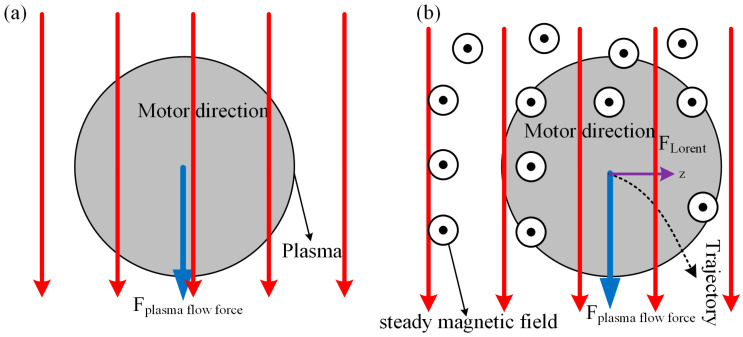
Schematic diagram of plasma forces: (**a**) plasma, and (**b**) the motion track of the magnetic field force on the plasma.

When the magnetic field acts on TIG welding, the maximum temperature of arc is close to tungsten pole. With increasing magnetic field, the temperature growth of arc near tungsten pole is gradually weakened, as illustrated in Figure 4a. At same time, the arc temperature decreases gradually along axial direction with increasing magnetic field. To explain this, an explanation is given in terms of the effect of the magnetic field on plasma, where the Lorentz force on electrons increases as the magnetic field strength increases, causing the plasma to deviate from the edge of the arc. Figure 5 shows diagrams of forces and trajectories of plasma. It can be seen from Figure 5 that the plasma is deflected to the right by the Lorentz force and temperature of arc depends on plasma. Therefore, the temperature on the arc axis decreases as the magnetic field strength increases.

**Figure 6:**
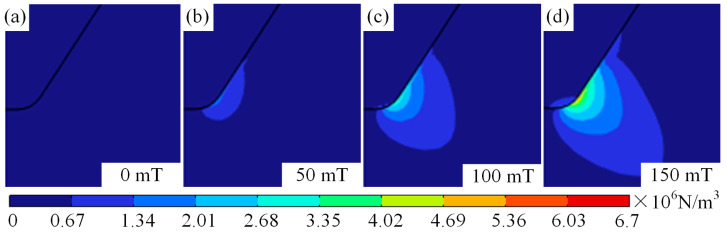
Force cloud of plasma under different magnetic fields: (**a**) magnetic field of 0 mT, (**b**) magnetic field of 50 mT, (**c**) magnetic field of 100 mT, and (**d**) magnetic field of 150 mT.

It should be noted that as the magnetic field changes, the form of temperature variation from centre to edge of the arc changes. The pattern transitions from a gradual centre-to-edge decrease to an initial increase followed by a decrease, as shown in Figure 4b–d. This change in the shape of the temperature field is mainly attributable to the deviation of plasma towards arc edges under magnetic field influence. Furthermore, it can be observed from Figure 4b–d that the variation in the arc temperature field induced by the magnetic field becomes increasingly pronounced with the increasing distance from the cathode. This is primarily due to the fact that as plasma moves further from the cathode, it deviates further from the arc axis under the influence of the Lorentz force. Figure 6 shows the force cloud of the plasma under different magnetic fields. With increasing magnetic field, the magnitude of the Lorentz force acting on plasma near the cathode gradually increases, and the area subject to the greater Lorentz force also gradually increases, as shown in Figure 6a–d. This results in plasma deviating further from the edge as the magnetic field strength increases, even though the distance travelled along the axis remains constant. This conclusion can also be verified by measuring the distance between the hot zone at the anode and the centre axis of the arc, as shown in Figure 3b–d. This is also one of the reasons why the temperature near the anode along the arc axis decreases as the auxiliary magnetic field strength increases.

### 3.2. Longitudinal Magnetic Field Effects on Velocity Field

**Figure 7:**
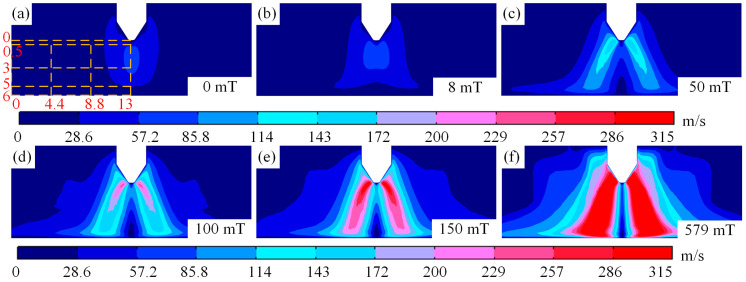
Motion velocity diagram of arc plasma at different magnetic fields: (**a**) magnetic field of 0 mT, (**b**) magnetic field of 8 mT, (**c**) magnetic field of 50 mT, (**d**) magnetic field of 100 mT, (**e**) magnetic field of 150 mT, and (**f**) magnetic field of 579 mT.

The velocity of arc plasma in different magnetic fields is illustrated in Figure 7. It can be observed from Figure 7a–d that when the magnetic field is low, the plasma velocity near the cathode is higher than that near the anode, and the plasma velocity in the arc edge is the lowest. When comparing the velocities of plasma near the cathode and anode, it is evident that although the velocity near the anode and arc edge is smaller, it increases with increasing magnetic field strength, as shown in Figure 7a–f. The smaller velocity of plasma near the arc edge can be explained by the accumulation of electrons. Electrons moving from the cathode to the anode lead to an increase in the number of electrons accumulated at the anode, and electrons repel each other. Therefore, as the number of electrons at the anode increases, the plasma velocity there is lower than that near the cathode. When the magnetic field strength exceeds 150 mT, the plasma velocity near the anode remains lower than that near the cathode. Then, the difference in velocity decreases, as shown in Figure 7e,f.

It is worth noting that as the magnetic field changes, the maximum velocity of the plasma arc gradually rises from 74 m/s at a magnetic field of 0 mT to 296 m/s at 150 mT. Meanwhile, a distinct low-velocity region appears near the arc’s central axis, which expands from the anode toward the cathode as the magnetic field increases, until a continuous low-velocity region forms along the central axis at a magnetic field of 579 mT, as shown in Figure 7b–f. This change in the plasma can be explained by the force exerted by the magnetic field on the plasma. When the magnetic field is small, the Lorentz force drags the plasma a short distance away from the central axis, resulting in higher plasma velocities near the central axis, as shown in Figure 7a,b. However, as the magnitude of the magnetic field grows, the plasma is dragged away from the arc axis by the Lorentz force, causing the plasma to move further away from the axis near the anode under the magnetic field is strong, as shown in Figure 7c–f.

**Figure 8:**
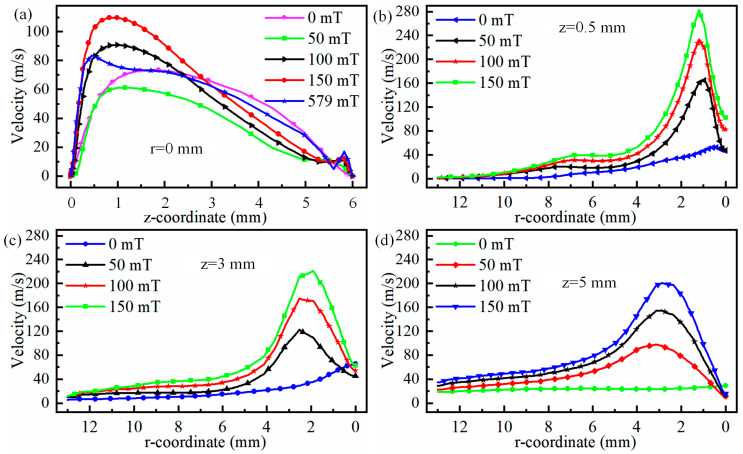
Variation in plasma radial velocity at 0.5 mm, 3 mm, and 5 mm positions below the cathode: (**a**) r = 0 mm, (**b**) z = 0.5 mm, (**c**) z = 3 mm, and (**d**) z = 5 mm.

The radial velocity of the plasma at positions 0.5 mm, 3 mm, and 5 mm below the cathode is illustrated in Figure 8. It can be observed from Figure 8a that regardless of the magnitude of the magnetic field, the plasma velocity along the central axis increases and then decreases from the cathode to the anode. Meanwhile, as the magnetic field strength increases, the maximum velocity along the central axis shifts closer to the cathode. Specifically, when the magnet field is 0 mT, the maximum velocity occurs at 1.6 mm below the cathode. When the magnetic field increases to 150 mT, the maximum velocity occurs at 0.8 mm below the cathode. Even when the magnetic field increases to 579 mT, the maximum velocity occurs at 0.5 mm below the cathode, as shown in Figure 8a. Meanwhile, regardless of the magnetic field, the plasma velocity at z = 6 mm is almost equal. The variation in plasma velocity along the cathode centre axis can be explained by the Lorentz force. When the plasma is near the cathode, the electric field force accelerates the plasma, but once it reaches a certain position, the electric field force begins to oppose the plasma velocity. In particular, near the anode, due to the accumulation of plasma, the opposing effect on the plasma moving toward the anode is strongest. Ultimately, when the plasma reaches the anode substrate at z = 6 mm, its velocity is reduced to zero under the combined effects of the electric field force and the substrate, as shown in Figure 8a. Additionally, the plasma along the central axis is deflected away from the central axis due to the magnetic field force. Therefore, the velocity at the central axis after applying the magnetic field is not the maximum velocity in the arc, as shown in Figure 8b–d.

**Figure 9:**
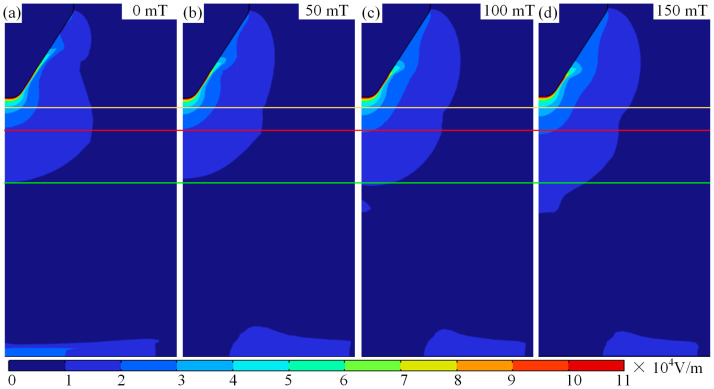
Electric field gradients of arcs in different magnetic fields: (**a**) magnetic field of 0 mT, (**b**) magnetic field of 50 mT, (**c**) magnetic field of 100 mT, and (**d**) magnetic field of 150 mT.

When observing the maximum velocity along centre axis in Figure 8a as a function of magnetic field, it is evident that as magnetic field becomes stronger, the maximum velocity along centre axis first reduces and then increases. This trend continues until a low-velocity region spanning both poles appears along centre axis, after which the velocity begins to reduce again as magnetic field strength becomes stronger. The reason for this change in velocity along centre axis can be explained by the electric field gradient. Figure 9 shows the electric field gradient of the arc under different magnetic fields. As shown in Figure 9a,b, when the magnetic field is 50 mT, the region with a larger electric field gradient is actually smaller than when the magnetic field is 0 mT. This also indicates that when the magnetic field is 50 mT, the acceleration of plasma along the central axis due to the electric field is smaller than when the magnetic field is 0 mT. When the magnetic field exceeds 50 mT, the region with a larger electric field gradient expands, leading to an increase in the acceleration distance of the plasma along the central axis. Therefore, when the magnetic field is less than 50 mT, the velocity along the central axis decreases, while it rises when the magnetic field strength exceeds 50 mT. It should be noted that when the magnetic field exceeds 579 mT, a low-speed zone appears along the central axis, meaning that the higher-energy plasma has completely deviated from the central axis. Therefore, the velocity along the central axis decreases at this point.

### 3.3. Longitudinal Magnetic Field Effects on Arc Pressure

**Figure 10:**
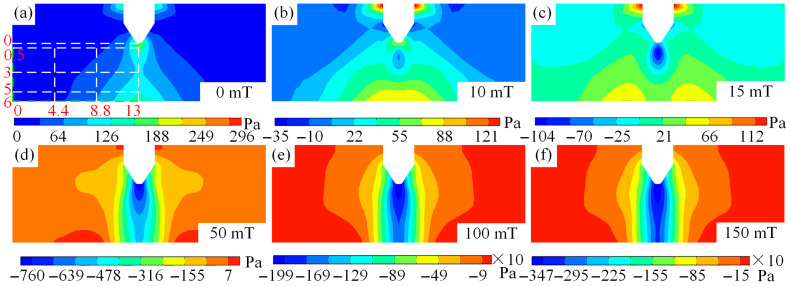
Arc pressure distribution of tungsten-pole TIG welding under different magnetic fields: (**a**) magnetic field of 0 mT, (**b**) magnetic field of 10 mT, (**c**) magnetic field of 15 mT, (**d**) magnetic field of 50 mT, (**e**) magnetic field of 100 mT, and (**f**) magnetic field of 150 mT.

The distribution of arc pressure in tungsten inert gas welding under different magnetic fields is illustrated in Figure 10. Figure 10a–f illustrates that when no auxiliary magnetic field is applied during arc welding, there is no negative pressure within the arc, and the arc pressure is relatively high at both the positive and negative electrodes, with positive values. However, when the auxiliary magnetic field is applied to TIG welding, negative pressure begins to appear inside the arc, and even after magnetic field is greater than 0.1 T positive pressure does not exist inside the arc. The arc negative pressure decreases from 296 Pa at a magnetic field of 0 T to −3470 Pa at 0.15 T, representing 12.72 times difference, as shown in Figure 10b–f. When TIG welding is performed without an external magnetic field, the absolute value of the cathode pressure is greater than that of the anode. When a magnetic field is applied, the absolute value of anode pressure becomes higher than that of cathode. Therefore, the auxiliary magnetic field provides a new method for regulating the pressure of the TIG welding pool. Notably, as the magnetic field is introduced, negative pressure first appears at the midpoint between the anode and cathode. With a magnetic field of 0.015 T, the pressure along the central axis of the anode and cathode is divided into two parts, and the pressure values of these two parts decrease as the magnetic field strength increases, as illustrated in Figure 10b–d. Additionally, as the magnetic field strength increases, the negative pressure first expands toward the cathode and then spreads across the entire central axis, as illustrated in Figure 10b–f. The appearance of arc negative pressure is primarily influenced by arc magnetostriction and thermal pressure.

**Figure 11:**
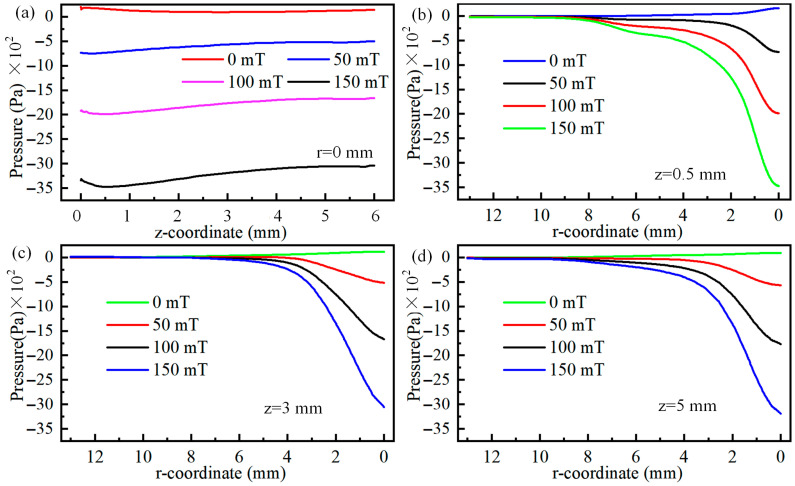
Pressure between the central axis and radial direction below the cathode: (**a**) r = 0 mm, (**b**) z = 0.5 mm, (**c**) z = 3 mm, and (**d**) z = 5 mm.

Figure 11 shows the plasma velocity at the arc centre axis and at positions 0.5 mm, 3 mm, and 5 mm below the cathode. As illustrated in Figure 11a, the arc pressure along the central axis exhibits an overall trend of first decreasing and then increasing, with the distance over which the arc pressure decreases being significantly smaller than that of increasing arc pressure. In other words, the arc pressure decreases rapidly while increasing slowly. Notably, it is observed that as magnetic field grows, the absolute values of arc negative pressure gradually increase, and the rate of decrease in negative pressure along the central axis becomes increasingly pronounced. Additionally, the upward trend in the negative pressure increase interval becomes increasingly pronounced as the magnetic field grows, illustrated in Figure 11a. Even the maximum pressure along the central axis decreases from 97 Pa at 0 T to −3038 Pa at 0.15 T, with a difference of up to 32.32 times between the two values, providing a reference for adjusting the pressure of the arc welding melt pool.

The absolute value of the arc radial pressure exhibits a trend of higher magnitudes in the centre and lower magnitudes at the edges, and its magnitude increases with increasing magnetic field strength, as shown in Figure 11b–d. When the centre position is 0.5 mm away from the cathode, the pressure value decreases from 164 Pa at 0 T to −3471 Pa at 0.15 T, with an absolute value difference of 22.16 times. As the arc distance from the cathode increases, the ratio of the maximum absolute values of pressure at 0 T and 0.15 T magnetic fields changes from 22.16 times at 0.5 mm from the cathode to 26.22 times at 3 mm, and further to 33.53 times at 5 mm. Arc pressure is related to the velocity of the plasma. As the magnetic field strength increases, the plasma velocity increases, leading to an increase in arc pressure. Additionally, radial force balance requires that the outward thermal pressure gradient must be balanced with the inward magnetic pressure gradient and the plasma’s own inertial force. This is why a noticeable negative pressure phenomenon occurs in the TIG welding arc after applying a magnetic field.

### 3.4. Longitudinal Magnetic Field Effects on Electric Field

**Figure 12:**
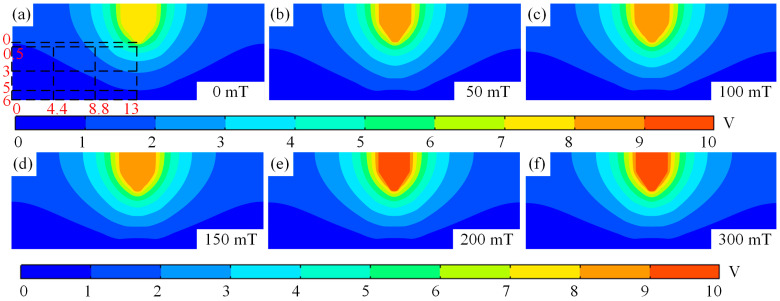
Cloud view of tungsten-pole TIG welding arc under different magnetic fields: (**a**) magnetic field of 0 mT, (**b**) magnetic field of 50 mT, (**c**) magnetic field of 100 mT, (**d**) magnetic field of 150 mT, (**e**) magnetic field of 200 mT, and (**f**) magnetic field of 300 mT.

Figure 12 shows the electric field contour map of the tungsten electrode TIG welding arc in different magnetic fields. From Figure 12a–f, as the magnetic field increases, as the magnetic field strength increases, the arc voltage increases only from 7.7 V at 0 T to 9.3 V at 0.3 T, indicating a minimal increase. Even when the magnetic field reaches 0.3 T, the voltage only increases by 1.6 V. Therefore, it can be concluded that the magnetic field has a negligible effect on the voltage. This is primarily due to the fact that after applying a magnetic field in TIG welding, the plasma shifts away from the central axis toward the periphery. This results in minimal disturbance to the electric field distribution, leading to insignificant voltage variations. Additionally, the arc voltage decreases gradually from the central axis towards the periphery, and the potential gradient beneath the cathode is larger than in other regions.

**Figure 13:**
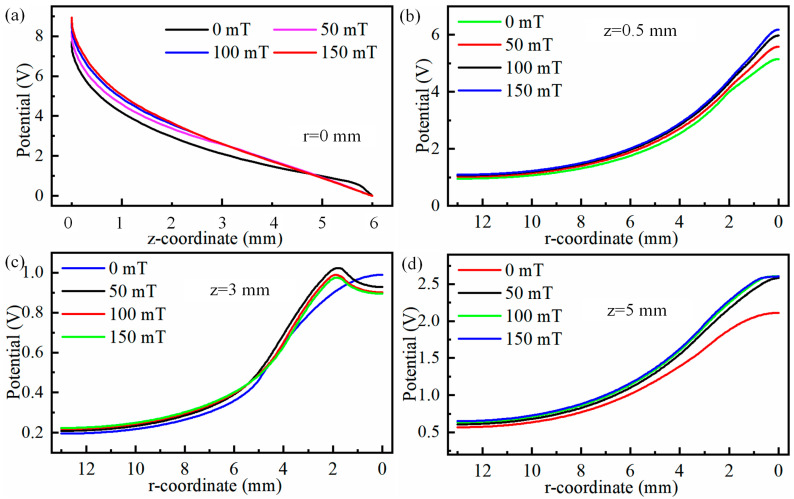
Radial potentials at centre axis of TIG weld and at 0.5 mm, 3 mm, and 5 mm positions below the cathode: (**a**) r = 0 mm, (**b**) z = 0.5 mm, (**c**) z = 3 mm, and (**d**) z = 5 mm.

Figure 13 shows the line graphs of the radial potential at the centre axis of the TIG weld and at positions 0.5 mm, 3 mm, and 5 mm below the cathode. From Figure 13a, it can be seen that the potential strength along the centre axis from the cathode to the anode initially decreases rapidly followed by a slower decrease, regardless of the auxiliary magnetic field. During the interval of rapid decrease in potential, the decreasing trend reduces slightly as the magnetic field strength increases, but during the slow decrease phase, the rate of decrease increases slightly as the magnetic field strength increases, as shown in Figure 13a. When radial potentials at 0.5 mm, 3 mm, and 5 mm positions below the cathode are observed, it is found that while the maximum potential at the 3 mm position below the cathode does not increase with increasing magnetic field strength, the potentials at 0.5 mm and 5 mm positions below the cathode increase with increasing magnetic field strength, as shown in Figure 13c,d. The maximum electric potential located 3 mm beneath the cathode does not increase with the rise in magnetic field strength. This phenomenon primarily occurs due to the deviation of the plasma from the central axis. Such deviation results in a reduction in the electric potential in the vicinity of the central axis region, as illustrated in Figure 12d–f.

## 4. Empirical Verification

**Figure 14:**
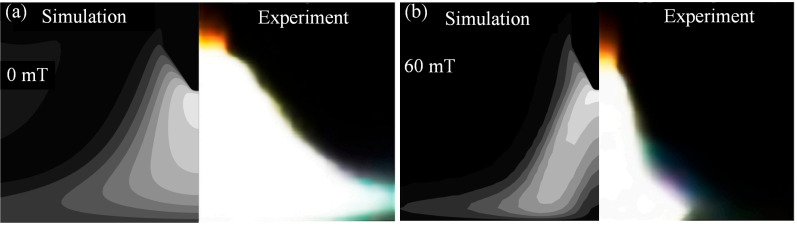
Experimental verification of arc shape: (**a**) comparison between simulation and experiment under the magnetic field of 0 mT, and (**b**) comparison between simulation and experiment under the magnetic field of 60 mT.

Due to experimental limitations, this paper uses the experimental results of Liu et al. to validate the correctness of the proposed model. During the experimental validation process, the arc current was set to 100 A, consistent with the settings of Liu et al. [25]. The arc shapes obtained from numerical calculations and experiments are shown in Figure 14. As can be seen from Figure 14a, when no magnetic field is applied, the arc shape exhibits a bell-shaped contour. After applying a magnetic field, the arc size is compressed, as shown in Figure 14b. Upon observing Figure 14, it is evident that although there are some discrepancies between the arc morphology calculated by the proposed model and the experimental arc morphology, the overall agreement is satisfactory. The primary reason for the discrepancies between the model and experimental results lies in the differences between the thermal-physical properties used in numerical calculations and those in actual conditions. Additionally, the numerical calculation process makes necessary assumptions about arc welding, which also differ from actual arc welding conditions. Therefore, the arc geometric shapes calculated by the proposed model differ from those in actual conditions.

## 5. Conclusions

(1) As the magnetic field increases, the arc morphology changes from bell-shaped to hollow, and the high-temperature region of the arc compresses and moves away from the arc centre axis. The maximum temperature of the arc decreases from 14,600 K at 0 mT to 13,500 K at 150 mT. With increasing auxiliary magnetic field strength, the high-temperature zone shifts toward the edge of the arc, with the maximum displacement of 0.45 mm occurring at 150 mT.
(2) The maximum plasma velocity of the arc occurs below the cathode regardless of whether a magnetic field is applied. The maximum velocity gradually increases from 74 m/s at 0 mT to 296 m/s at 150 mT. In addition, a clear low velocity zone appears along the central axis of the arc. This zone gradually expands from the anode toward the cathode as the magnetic field strength increases. At a magnetic field strength of 579 mT, this low-velocity zone extends through the entire central axis region between the cathode and the anode.
(3) When the magnetic field strength is 0 mT, the pressure near the cathode and anode of the arc is larger, and the middle area is smaller. As the magnetic field strength increases, negative pressure first appears near the cathode. This negative pressure region then expands toward the cathode with increasing magnetic field strength, subsequently extending toward the anode, until a distinct negative pressure zone emerges along the entire central axis. During the process of negative pressure expansion, the arc pressure decreases from 296 Pa at 0 mT to −3470 Pa at 150 mT, with a difference of 12.72 times.

## Data Availability

The original contributions presented in this study are included in the article. Further inquiries can be directed to the corresponding authors.

## Glossary

z	Axial coordinates
P	Gas pressure
$c_{p}$	Heat capacity
$F_{r}$	r-direction volumetric forces
J	Current density
g	Acceleration of gravity
$F_{lorent}$	Electromagnetic force
V	Plasma velocity
a	Acceleration of plasma
σ	Argon conductivity
μ0	Vacuum permeability
T	Temperature of argon
TR	Temperature of radiating body
u	Axial velocity
v	Radial velocity
μ	Argon viscosity
k	Thermal conductivity
$F_{z}$	z-direction volumetric forces
B	Magnetic intensity
Q	Energy
$B_{1}$	Longitudinal magnetic field
Jr	Radial current density
φ	Electric potential
Jz	Axial current density
K	Boltzmann’s constant
e	Electron charge
r	Radial coordinates
